# Mortality among 5-17 year old children in Kenya

**DOI:** 10.11604/pamj.2017.27.121.10727

**Published:** 2017-06-15

**Authors:** Bonface Ombaba Osano, Fred Were, Shanaaz Mathews

**Affiliations:** 1 University of Nairobi, College of Health Sciences, Kenya; 2 University of Cape Town, South Africa

**Keywords:** Kenya, mortality, children, teenage, adolescent

## Abstract

**Introduction:**

Global mortality trends have changed over time and are expected to continue changing with a reduction in communicable diseases and an increase of non-communicable disease. Increased survival of children beyond five years may change mortality patterns for these children. There are few studies in Africa that explore the causes of mortality in children over five years. The objective of this study was to determine the mortality rate and clinical profiles of children aged 5-17 years who died in six Kenyan hospitals in 2013.

**Methods:**

Retrospective review of patients’ medical records to abstract data on diagnosis for those who died in year 2013. Data was analysed to provide descriptive statistics and explored differences in mortality rates between age groups and gender.

**Results:**

We retrieved 4,520 patient records. The in-hospital mortality rate was 3.5% (95%CI 3.0-4.1) with variations in deaths between the ages and gender. Among the deaths, 60% suffered from communicable diseases, maternal and nutritional causes; 41.3% suffered from non-communicable diseases. A further 11.9% succumbed to traumatic injuries. The predominant clinical diagnoses among patients who died were HIV/AIDS, respiratory tract infections and malaria.

**Conclusion:**

Infectious causes had the highest proportion of diagnoses among children aged 5-17 years who died.

## Introduction

Globally, mortality trends have changed over time and are expected to continue changing with an increase in injuries, mental illness, drug abuse and poisoning [1–3]. The burden of disease studies have shown that communicable diseases are the leading cause of global mortality, with a transition underway as non-communicable diseases are on the rise [1]. There is great variation in causes of mortality between regions, age groups and gender [2, 3]. Global burden of disease studies show that mortality rates increase from 15 years for females and 20 years for males [2, 4–6]. In developed countries, non-communicable diseases cause greater morbidity and mortality in children than communicable diseases [1–3, 7]. Although the causes of mortality are shifting to non-communicable causes, the burden of communicable disease remains significant in middle- and low-income countries where communicable diseases still account for the majority of mortality cases [1–3, 7]. When causes of mortality are analysed in low income countries, reproductive health issues (pregnancy and maternity related illnesses) dominate in females as they become older, whereas injuries are a leading cause of mortality in older males [1, 3]. The main causes of death in Sub-Saharan Africa are preventable diseases caused by infectious agents [8–11]. Communicable diseases have been the leading cause of mortality and still remain so in Sub-Saharan Africa, unlike in developed countries where non-communicable causes are on the increase [2, 12–14]. These communicable diseases include infections like malaria, HIV/AIDS, pneumonia, tuberculosis (TB) and diarrhoea, especially for younger children [2, 9, 15]. The communicable disease may be leading probably due to co-morbidities such as malnutrition and HIV. In Kenya, for example, up to 11% of children below 5 years are underweight and a higher fraction (2^6^%) are stunted [16, 17]. These levels of malnutrition may be prevalent in the older children and thus contribute to the mortality from infectious causes. Globally, complications from pregnancy among teenagers contribute to mortality among adolescents [2]. This is mainly due to teenage pregnancies as a consequences of early marriages in the context of developing countries such as in Africa where cultural practices impact on health outcomes [18]. The large proportion of girls under 18 years who are pregnant may contribute to the high maternal mortality in Sub-Saharan Africa [16, 19]. Non-communicable diseases, mental illness and substance abuse also play a role in adolescent mortality in Africa; however very little is known about their contribution to mortality in developing countries as they are not well documented as in the high-income countries [2].

Injuries as a cause of mortality are expected to rise with increasing economic prosperity resulting in epidemiological transition and a decrease in communicable diseases, more so in developing countries [1]. Intentional and unintentional injuries, such as assault, burns and road traffic accidents (RTA) are a major cause of mortality among adolescents and young adults aged 15-24 years, and especially among males in Kenya [12], other low-income countries [13] and globally [2, 5, 11, 20, 21]. Suicide is one of the leading causes of death among adolescents in the United States of America and other high-income countries [2, 20]. In most regions of the world, suicide is more common in males than females [2, 20, 22]. Outdoor injuries from violence are more common amongst males, while in females indoor injuries, such as burns, are slightly more common [13]. Cyclists and pedestrians are more likely to be killed than car occupants [23, 24]. Road traffic accidents are an emerging public health problem, especially from motorcycles in Tanzania [25]. In South Africa, Balme, et al found poisoning to be a cause of childhood hospital admission. Accidental poisoning may be more common among the younger age groups but intentional poisoning (suicide attempts) as well as homicide may be more common with the older groups. Pesticides and household cleaning products have also increased as causative agents of poisoning [26]. There is limited data available on children older than 15 years in Kenya, as they have not been explored as a group on their own and are instead grouped with adults or missed completely. In addition, there are few studies on burden of disease based on Africa with a focus on children older than five [2, 5, 27]. Civil registration of deaths is unreliable due to under-reporting and misclassification, with ill-defined incorrect ICD-10 codes used in some instances [10, 28]. To inform appropriate prevention strategies it is necessary to know the pattern of causes and magnitude of deaths across all ages [5]. There is little information in Kenya on the exact causes of death among 5-17 years of age yet they have been shown to contribute to close to 10% of deaths. In Kenya, close to 8% of deaths that occurred in 2003-2009 in a rural area of the western region were in late adolescents (15-19 years) and young adults (20-24 years) [12]. Preventable infectious causes, such as malaria, lower respiratory tract infections, TB, HIV, malaria and gastroenteritis, account for a large proportion of mortality in Kenya as in the rest of Africa [2, 29, 30]. Even though infectious diseases are the leading cause of mortality in Kenya, the exact infectious causes have varied over time and by age [3, 9, 10, 12]. There seems to be a reduction of up to 60% in mortality from gastroenteritis and HIV [12, 10]. With increased survival of children under five years of age, there are likely to be an increase in children aged 6-18 in the near future and there is an urgent need to understand their health issues better. The Kenyan Demographic and Health Survey of 2008-2009 showed that children aged 5-19 years comprised 39% of the Kenyan population; yet there is little information on the health of this age group [16]. This study aimed to determine the in-hospital mortality rate, diagnoses at death of children aged 5-17 years admitted to six hospitals in Kenya for the 2013 calendar year and to explore pattern of mortality by age.

## Methods

The study was carried out in five government hospitals spread across different regions of Kenya and one private hospital in Nairobi. The five government hospitals were purposively selected because they are the referral hospitals for the counties they serve and represent a wide geographical area within the country. The private hospital was included to provide a perspective of children from a higher socio-economic group. A retrospective hospital based study using admission registers as the sampling frame, for the 2013 calendar year was used to identify all children aged 5-17 years. The study included all children aged 5-17 as the study sample. Hospital records were retrieved, reviewed and data abstracted from patient’s discharge or death summaries for interest variables (age, gender and diagnosis at death). The researcher or trained research assistants reviewed the whole patient record to extract the required data where there were no summaries. Three patients’ records from the private hospital were not available for review due to medico-legal reasons and were excluded from this analysis. In this study, age was categorised into groups (5-9, 10-14 and 15-17 years) as a modification of the age categories used by Lozano [3], Patton [2], Wang [10], and the Kenya Demographic and Heath Survey [16] with the upper age cut-off of 17 years as the legal age cut-off for children. The mortality proportion was calculated as all the deaths in the age group divided by the age group total admissions, expressed as a percentage to describe the mortality pattern. Pearson’s chi-square test and odds ratios were calculated to explore differences in deaths between genders, age groups and HIV status at 95% confidence interval. The patients’ diagnoses were categorised in accordance with the Lozano [3] study classifications and to allow for comparison.

### Classification of diseases

The disease classification used was based on the study by Lozano, et al on global and regional mortality from 235 causes of death for 20 age groups in 1990 and 2010; a systematic analysis for the Global Burden of Disease (GBD) Study [3] as shown in Table 1. This classification was chosen to make comparison of findings with other international studies easier. However, many patients had multiple diagnoses with some appearing across disease classification categories and hence some patients fell in more than one disease category. Patients with injuries did not have full details on where it occurred, the cause, nature and type of injury and were therefore grouped together as injuries. We obtained approval from the Human Research Ethics Committee at the Faculty of Health Sciences, University of Cape Town (UCT) and the Kenyatta National Hospital/University of Nairobi Ethics and Research Committee (KNH-UoN ERC). Hospital review boards and administration permission were obtained from all the hospitals where the study was conducted. Patient confidentiality was maintained at all times. Names of the patients/study participants were not collected by the study. Once the hospital number was noted, it was de-identified by giving it a code that could not be traced back to the patients. Only coded data was transported to conceal identities. In addition, the data was stored in files kept under lock and key with restricted access in secured offices. Electronic databases were encrypted and stored in password-protected, secure computers.

**Table 1:** Classification of diseases as adopted from Lozano et al. (2010). Global and regional mortality from 235 causes of death for 20 age groups in 1990 and 2010; a systematic analysis for the GBD Study [3]

Group 1: communicable, maternal, neonatal, and nutritional disorders	Group 2: non-communicable diseases (NCDs)	Group 3: injuries
A. Infections, such as HIV/AIDS, diarrhoea, meningitis, and other common infectious diseases. B. Respiratory infections. C. Maternal causes: normal pregnancy with delivery and pregnancy-related illnesses, such as ante-partum haemorrhage, abortion, post-partum haemorrhage, puerperal sepsis and maternal mortality. D. Perinatal/neonatal disorders. E. Nutritional deficiencies.	A. Neoplasms. B. Haematological disorders and other neoplasms. C. Diabetes. D. Endocrine disorders. E. Neuropsychiatric conditions. F. Sense organ diseases, such as eye and ear, nose and throat (ENT). G. Cardiovascular diseases. H. Chronic respiratory diseases, such as asthma. I. Digestive diseases, such as liver disease. J. Genitourinary diseases. K. Skin diseases. L. Musculoskeletal diseases M. Congenital anomalies. N. Oral conditions.	A. Unintentional injuries. i. Road traffic accidents. ii. Poisonings. iii. Falls. iv. Fires. v. Drowning. vi. Other unintentional injuries. B. Self-harm and interpersonal violence. i. Self-inflicted injuries. ii. Violence. iii. War and legal intervention. iv. Other intentional injuries. C. Forces of nature.

## Results

There were 160 deaths in the age group 5-17 years out of 4,520 total admissions at the six hospitals in the study population. The in-hospital mortality rate was 3.5% (95% CI, 3.0 – 4.1%) as shown in Table 2. The deaths of 5-17 year olds contributed 3.9% of all age groups hospitals’ total deaths. The non-communicable causes had the highest category-specific mortality proportion of 4.4% while both communicable diseases and injuries were at 3.0%. Most of the deaths occurred in younger children aged 5-9 years of age (40.8%), although the age specific mortality proportion was highest in the age group 10-14 years. Those aged 15-17 years had the best outcomes with a 2.3% in-hospital mortality rate while those aged 10-14 years had the worst outcome, with a 5.0% in-hospital mortality rate (Table 2). There were significantly (p<0.001) more male deaths (90 - 57%) compared to female deaths (67-43%).

**Table 2:** Deaths by age group and gender

Age group	5-9 years	10-14 years	15-17 years	Total
Deaths	64	56	37	160^+^
Total admissions for age group	1 808	1 111	1 601	4 520
In-hospital mortality rate (95% CI)	3.5%(2.7-4.5)	5.0%(3.8-6.5)	2.3%(1.6-3.2)	3.5%(3.0-4.1)
**Gender**	**Male**	**Female**	**P value**	
Number (Percentage)	90(57%)	67(43%)	<0.001	160*

+ Includes three patients whose records could not be obtained.

### Diagnosis of patients who died

Table 3 shows disease classification of children aged 5-17 years who died at the six hospitals in 2013. Overall communicable causes were the main cause of death (60%). Table 4 shows the clinical diagnosis for the patients who died whereby infectious diseases such as HIV, malaria and pneumonia were the leading cause of death. There were four maternal deaths; three were from obstetric haemorrhage while one was from severe eclampsia. Poisoning contributed to 42% of deaths from injuries, while head injuries contributed 10.5%; and fractures and soft tissue injuries contributed 21.3% of injury patient deaths. The leading diagnoses in those who died were infectious (HIV, respiratory tract infections and malaria), haematological or injuries. Table 4 gives the clinical diagnosis of the patients who died. Some patients who died had multiple diagnoses across disease categories; hence disease categories are cumulative and exceed the number of deaths.

**Table 3:** Diagnosis of patients who died (number, percentage and category-specific in-hospital mortality rate for 5-17 year olds)

Causes	Category 1 (Communicable diseases, maternal and nutritional)	Category 2 (Non-communicable diseases)	Category 3 (Injuries)	Total
Number of deaths of 5-17 year olds from the category diseases	96	66	19	160^+^
Percentage contribution of category diseases’ deaths to all 5-17 year old in-hospital deaths	60.0%	41.3%	11.9%	100.0%
Category in-hospital mortality rate (deaths in category as a percentage of category admissions)	3.0%	4.4%	3.0%	3.5%
Number of admissions of 5-17 year olds from the category diseases	3 241	1 495	630	4 520

+ Includes three patients whose records could not be obtained.

**Table 4:** Clinical diagnosis for the 160 patients who died

Clinical diagnosis at death	Number of patients	Percentage of total deaths
HIV/AIDS	29	18.10%
Malaria	27	16.90%
Anaemia	26	16.30%
Pneumonia	24	15.00%
Meningitis/encephalitis	17	10.60%
Tuberculosis	12	7.50%
Diarrhoea/Gastroenteritis	11	6.90%
Other injuries including burns	11	7.00%
Poisoning and/or suicide	8	5.00%
Sickle cell disease	7	4.40%
Tumours & Leukaemia	7	4.40%
Rheumatic fever and other heart diseases	6	3.80%
Intestinal obstruction	6	3.80%
Malnutrition	6	3.80%
Liver disease/Hepatitis	5	3.10%
Congenital heart disease	4	2.50%
Obstetric causes (APH/PPH/eclampsia)	4	2.50%
Sepsis/abscess	3	1.90%
Cerebrovascular accident	3	1.90%
Periodontal disease	2	1.30%
Leishmaniasis	1	0.60%
Epilepsy/convulsive disorders	1	0.60%
Diabetes	1	0.60%
Schizophrenia	1	0.60%

## Discussion

We found that the proportion of deaths among 5-17 year olds in relation to total hospital deaths (3.9%) is lower than the age group’s contribution to overall hospital admissions (6.9%). This was lower than in a study in a rural area of the western region in Kenya where close to 8% of deaths were among adolescents aged 15-19 years and young adults aged 20-24 years [12]. These studies are not directly comparable since the one in Western Kenya included young adults where mortality rates are expected to start rising based on the findings from global burden of disease studies [3, 21]. The Phillips-Howard study in Western Kenya drew on a sample aged 15-24 years and therefore a direct comparison is not possible. In addition, the western part of Kenya has a different disease pattern, such as higher rates of malaria than the regions included in this study [12]. This study also found significantly more male than female deaths. This may be due to the fact that females were admitted mainly due to communicable diseases and maternity-related causes which may be appropriately managed at this level. Communicable, maternal and nutritional causes were diagnosed in over 60% of patients who died with the leading ones being malaria (17.2% of deaths), HIV/AIDS (18.1% of deaths) and pneumonia (15.0% of deaths). The major diagnoses at death in this study are the common causes of death in prior studies in similar settings [2, 8–14, 29, 30]. These are all communicable and preventable disease but due to social conditions and inequitable access to health care and services we still have large number of children dying from preventable causes. Poor access and health seeking behaviour, where patients come to formal healthcare facilities when it is very late when illnesses have complicated and probably more difficult to cure, may contribute to a high number of deaths from preventable and curable illnesses. Anaemia was one of the leading diagnoses in patients who died. The anaemia may be considered nutritional or a complication of the infectious causes such as malaria. There were four maternal deaths and this may have been contributed to by the young age of these mothers. Young maternal age is considered a risk factor for poor pregnancy outcomes for both mother and baby [31, 32]. The proportion of 5-19 year old females who have started child bearing is higher than that found in prior studies and surveys.

In Kenya 17.7% of women aged 15-19 have begun child bearing [16, 19]. This figure increased slightly to 18% as per the 2013 KDHS [17]. In addition to the young child bearing age being a risk factor, reproductive health services may not be adolescent friendly or easily accessible to these children thus worsening the outcomes. The non-communicable diseases were diagnosed in 41.3% of the overall deaths. This is in keeping with increases of NCDs as a cause of death in developing countries probably due to improvements in the socio-economic status [1–3, 12]. Better socio-economic status may shift morbidity and mortality from infectious causes to non-communicable disease and injuries as seen in more developed countries [1–4]. Injuries attributed to 11.9% of deaths. Males were more likely to die due to injuries but this difference was not statistically significant. The lack of a gender difference in injury deaths in this study was probably because the injuries were analysed as grouped and were not specified by type or cause. The limitations of incomplete documentation of exact nature of injury (interpersonal violence, intentional or unintentional) could not allow for separation to analyse this difference further. Some injury types, such as interpersonal violence, may increase with age and are more common in males. The changes in causes of mortality to non-communicable diseases that have been noted in developed countries were not reflected in this study. Although injuries and non-communicable diseases were documented, communicable diseases were still the leading cause of death in Kenya for children aged 5-17 years as in most other low income countries [2, 8–14, 29, 30].

### Limitations

This study depended on hospital records, which did not have full documentation for all patients. The gap in information was supplemented by reviewing the full patient records, inferring from the investigations done and the management plan for each of those patients. The diagnoses at death may have been multiple for each death and not the immediate cause of death could be specifically ascribed. Mortality rate was not calculated because of the unavailability of population data for each district and the catchment areas.

## Conclusion

Children aged 5-17 years contribute 3.5% of all in hospital deaths. Infectious and maternal causes were the leading cause of hospital deaths (60%). The deaths were mainly from preventable and curable diseases, which should not be causing mortality with appropriate preventive and curative services. This calls for strengthening of prevention of the non-communicable and common infectious causes in this age group. This could be achieved through the school health programme because almost all of these children are in school and it is the easiest place to reach them.

### What is known about this topic

- Communicable diseases are a leading cause of death in sub-Saharan Africa;
- Injuries and non-communicable causes of mortality are rising.

### What this study adds

- Communicable causes are the leading causes of death in children aged 5-17 years in Kenya;
- The deaths were mainly from preventable and curable diseases, which should not be causing mortality with appropriate preventive and curative services.

## Competing interests

The authors declare no competing interest.
